# Investigation of the Softening Resistance Behavior and Its Mechanism in Cu-Ni-Si Alloys with Discontinuous Precipitates

**DOI:** 10.3390/ma17215323

**Published:** 2024-10-31

**Authors:** Yicheng Cao, Wei Luo, Zhen Yang, Haofeng Xie, Wenjing Zhang, Zengde Li, Lijun Peng, Yunqing Zhu, Jun Liu

**Affiliations:** 1 State Key Laboratory of Nonferrous Metals and Processes, GRINM Group Co., Ltd., Beijing 100088, China; caoyicheng1995@163.com (Y.C.);; 2 GRIMAT Engineering Institute Co., Ltd., Beijing 101407, China; 3 General Research Institute for Nonferrous Metals, Beijing 100088, China

**Keywords:** automotive connectors, Cu-Ni-Si alloy, discontinuous precipitation, soften resistance, soften mechanism

## Abstract

In this study, isothermal annealing experiments were conducted on copper-nickel-silicon alloys containing continuous precipitates (CP) and discontinuous precipitates (DP) to investigate the effects of different types of precipitate phases on the microstructural evolution and softening temperature during annealing, as well as to analyze the differences in softening mechanisms. The experimental results revealed that the softening temperature of the CP alloy, subjected to 75% cold deformation, was 505 °C. In contrast, the DP alloy achieved softening temperatures of 575 °C and 515 °C after 75% and 97.5% cold deformation, respectively. This indicates that the DP alloy exhibits significantly superior softening resistance compared to the CP alloy, attributed to the distinct softening mechanisms of the two alloys. In the CP alloy, softening is primarily influenced by factors such as the coarsening of the precipitate phase, the occurrence of recrystallization, and the reduction in dislocation density. In the DP alloy, the balling phenomenon of the DP phase is more pronounced, and its unique microstructure exerts a stronger hindrance to dislocation and grain boundary motion. This hindrance effect reduces the extent of recrystallization and results in a smaller decrease in dislocation density. In summary, the DP alloy, due to its unique microstructure and softening mechanisms, demonstrates better softening resistance, providing higher durability and stability for high-temperature applications.

## 1. Introduction

In recent years, the rapid development of new energy vehicles has significantly driven the miniaturization, high current, and high voltage trends in components such as onboard chargers, DC charging piles, and other automotive parts. Among these, high-current connectors play an irreplaceable role in these devices [1,2]. Automotive connectors are essential components in electronic systems, responsible for power transmission, signal control, and communication [3]. They primarily consist of terminals, insulators, housings, and accessories, with the terminals being the core components that facilitate electrical contact within the connectors [4].

Among various electronic and electrical materials, copper alloys stand out as the most widely used materials for connector terminals due to their excellent electrical conductivity, tunable strength, and superior elasticity [5]. Copper is also critical to the automotive industry, especially for electric vehicles (EVs), where demand is expected to increase significantly. For example, according to the International Copper Association, by 2040, copper demand for EV components will rise to 6 million tons annually, representing a 143% increase from 2020. The main driver of this growth is the fact that by 2040, an electric vehicle will require approximately 73 kg of copper, compared to just 30 kg in a traditional internal combustion engine vehicle. Key components such as electric traction motors, lithium-ion batteries, and low-voltage wiring harnesses will account for the largest sources of copper demand [6,7]. This surge in demand highlights the importance of optimizing the properties of copper alloys used in high-current connectors to meet the growing needs of the automotive industry.

The performance requirements for electrical connectors include high contact force, minimal deformation during displacement, small bending angles, resistance to failure under cyclic stress, low contact resistance, good thermal conductivity, low temperature rise, and the ability to operate for extended periods in high-temperature environments [8,9,10,11,12]. Therefore, copper alloys used in electrical connectors must possess high tensile strength, good elastic modulus, high electrical conductivity, a high thermal conductivity coefficient, and excellent resistance to high-temperature softening [13,14,15,16,17,18,19,20,21,22].

Cu-Ni-Si alloys are widely used in the manufacturing of electrical connectors, thanks to their excellent combination of mechanical strength and electrical conductivity. These properties are primarily achieved through precipitation strengthening, wherein nanoscale Ni_x_Si precipitates form during the solid solution and aging treatments. These precipitates provide a strong strengthening effect, enhancing the alloy’s overall performance. Additionally, after aging, further improvements in strength and conductivity can be achieved through cold working [23]. In Cu-Ni-Si alloys, Ni₂Si is the primary precipitate formed during the aging process. However, under certain conditions, such as rapid cooling following a high-temperature solution treatment or shorter aging times, Ni₃Si-rich zones may also form. Ni₃Si phases tend to precipitate at higher temperatures (above 600 °C) or with prolonged heat treatments. In alloys with compositions such as Cu-4.75Ni-1.13Si and a Ni/Si atomic ratio of 2, Ni₂Si formation is favored. Studies have consistently shown that both continuous and discontinuous precipitates in these compositions are predominantly Ni₂Si phases [24,25,26,27].

In numerous studies [28,29,30,31,32,33,34,35,36,37], to significantly enhance the strength of Cu-Ni-Si alloys by precipitating a large number of fine Ni_2_Si phases, shorter aging times are often employed to ensure precipitation. However, with shorter aging times, some Ni and Si atoms remain in solid solution within the Cu matrix and do not precipitate, which inevitably sacrifices the alloy’s electrical conductivity. This phenomenon also limits the further simultaneous improvement of both strength and conductivity in Cu-Ni-Si alloys.

Han et al. [26,38,39,40,41,42] indicated that by altering the type of precipitated phase in Cu-Ni-Si alloys specifically, through over-aging techniques, large amounts of nanoscale disc-shaped continuous precipitates can be transformed into fibrous discontinuous precipitates with thicknesses of several tens of nanometers and lengths of several micrometers. Due to the coarser size of these precipitates and the longer aging time, the alloy in the aged state exhibits lower strength and higher electrical conductivity.

Subjecting the aged alloy to large plastic deformation (75–97.5% area reduction) allows the coarser discontinuous precipitates to undergo plastic deformation under stress, further reducing their thickness to a few nanometers and significantly decreasing the internal spacing. This enhances the contribution of the precipitate strength, and the initially disordered arrangement of the precipitates becomes ordered along the direction of deformation under stress [43,44,45,46,47]. This reorientation weakens the scattering effect of the precipitate interfaces on electrons, thereby further improving the alloy’s electrical conductivity. Upon achieving a deformation of 97.5%, the alloy can simultaneously reach a strength of 1000 MPa and an electrical conductivity of up to 50% IACS.

Cu-Ni-Si alloys with discontinuous precipitates could be used as high-current connectors due to their high performance, and heating can be one of the main factors affecting connector service life under these circumstances. When connectors are working at high temperatures for long durations of time, the alloy may soften, resulting in a decrease in the mechanical properties of the alloy, thus affecting the reliability and life of the device [5,48,49]. Therefore, there is a need to improve the softening resistance of the alloys to ensure their stability when working in high-temperature environments. In traditional Cu-Ni-Si alloys, the main factors leading to the softening of the alloys are restitution, recrystallization, grain growth, coarsening of precipitates, reduction of dislocation density, etc. DP-reinforced Cu-Ni-Si alloys, as a new type of alloy, have not been investigated by many researchers in terms of their softening resistance, and their softening mechanism has not been clearly explained. Hence, it is necessary to carry out an in-depth study.

In this study, Cu-4.75Ni-1.13Si alloy was used to investigate the effects of different kinds of precipitates on the microstructure, mechanical properties, electrical conductivity, and anti-softening properties of the alloy. The anti-softening temperature of the Cu-Ni-1.13Si alloy containing discontinuous precipitates is given, and the anti-softening mechanism of the alloy is elucidated.

## 2. Experimental Procedure

**Figure 1:**
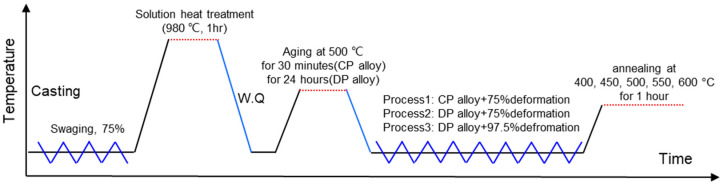
The roadmap of alloy processing.

The ingots of Cu-4.75Ni-1.13Si alloy in wt.% were prepared by induction melting using pure Cu (99.99%, Tianjin Huarui New Material Technology Co., Tianjin, China), pure Ni (99.9%, Tianjin Huarui New Material Technology Co., Tianjin, China), and pure Si (99.9%, Tianjin Huarui New Material Technology Co., Tianjin, China). The ingots were homogenized at 900 °C for 4 h, then hot extruded from φ32 mm to φ10 mm at 830 °C. The extruded rods were subsequently water quenched after being solution heat treated at 980 °C for 1 h. The quenched rods were aged at 500 °C for 30 min and 24 h to obtain alloys with only CP microstructure or DP microstructure, respectively. The CP and DP alloys were then subjected to different levels of area reduction at room temperature, with the CP alloys reduced by 75% area reduction (defined as Process 1), the DP alloys by 75% area reduction (defined as Process 2), and the DP alloys by 97.5% area reduction (defined as Process 3), as illustrated in the process roadmap in Figure 1. It was observed that when the area reduction exceeded 75%, the CP alloys tended to fracture during the drawing process, indicating that the DP alloys possess superior cold workability compared to the CP alloys.

The Vickers hardness was measured using a hardness testing machine (WILSON VH1150, ITW Company, Shanghai, China), with a 500 g load and 15 s loading time. All specimens were tested over 10 times, and their values were averaged. The Intelligent DC Low Resistance Tester (TH2512B, RPE Company, Suzhou, China) was used to measure the electrical conductivity. The environmental temperature is about 20 °C, and the gauge length is 300 mm. The tensile tests were conducted on dumbbell and wire samples using a universal testing machine (CMT7000 50KN, SANS, Shenzhen, China). Dumbbell and wire samples, with a gauge length of 25 mm, were subjected to tensile testing at a strain rate of 1/s. At least three parallel specimens were selected for each sample type.

The microstructure of the alloys was observed using a JSM-7900F field emission scanning electron microscope (FE-SEM, JSM-7900F, JEOL Company, Tokyo, Japan) at an accelerating voltage of 30 kV. The specimens were prepared by mechanical polishing and then electrochemical polishing in a 40% phosphoric acid solution. The recrystallization organizations of annealed alloys were characterized using EDAX-TSL electron-backscattered diffraction (EBSD, EDAX-TSL, Pleasanton, CA, USA). The samples were mechanically polished and then vibratory polished. Transmission electron microscopy (TEM, Talos F200X, FEI Company, Hillsboro, OR, USA) was used to observe the size and evolution behavior of precipitation. The TEM sample was stamped into 3 mm discs after being thinned to 50 μm thick and then prepared using double jet electropolishing and argon ion milling. X-ray diffraction (XRD, D/max 2550, Rigaku Corporation, Tokyo, Japan) analysis was performed with a scanning angle of 30° to 120° and a scanning speed of 3°/min. The experimental data were analyzed using MDI-Jade software (version 5.0, Materials Data Inc., Livermore, CA, USA) then used to calculate the density of dislocation by the Modified Williamson-Hall method.

## 3. Results

**Figure 2:**
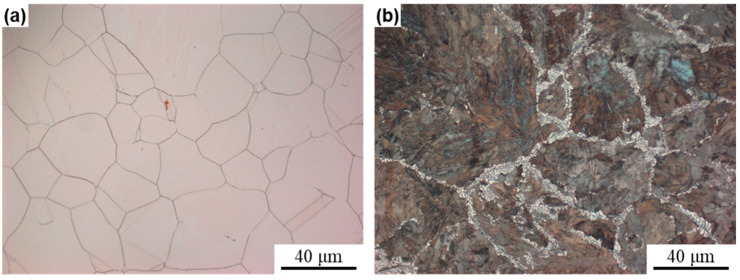
Optical images of CP alloy (**a**) and DP alloy (**b**).

Figure 2 shows the microstructure of Cu-4.75Ni-1.13Si alloys aged at 500 °C for 30 min and 1440 min. As can be seen from the figure, there is no DP found in the CP alloy, whereas the grains in the DP alloy are fully occupied by DP. In addition, due to the prolonged aging time, the grain size of the DP alloy is larger than that of the CP alloy. This design allows a good distinction between the effects of the different types of precipitates on the softening properties of the alloy. Comparison of the properties of the two alloys is shown in Table 1, from which it can be seen that the DP alloy has higher electrical conductivity compared to the CP alloy, but its strength is slightly lower than that of the CP alloy, and both alloys have low strengths due to the lack of cold deformation treatment, which is consistent with the previously reported studies.

**Figure 3:**
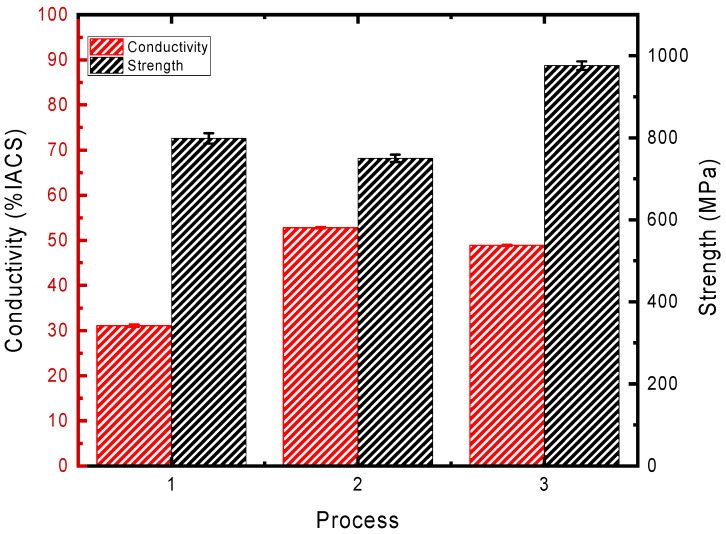
Strength and conductivity of the alloys following three different deformation processes.

Due to the fact that DP alloys exhibit superior mechanical and electrical properties only after cold deformation, it is essential to investigate the influence of DP on the alloy’s softening resistance by subjecting alloys with different types of precipitates to cold deformation, as illustrated in Figure 1. The mechanical and electrical properties of the alloys following three different deformation processes are summarized in Figure 3. It can be observed from the table that, at the same strain, DP alloys exhibit a lower strength (approximately a 6% reduction) but possess significantly higher electrical conductivity compared to CP alloys (approximately a 70% increase). As the degree of deformation of the DP alloys further increases (Process 3), the strength of the alloys continues to rise while the electrical conductivity experiences only a slight decline, remaining considerably higher than that of the CP alloys. This excellent combination of properties allows DP alloys to be utilized in a broader range of applications.

**Figure 4:**
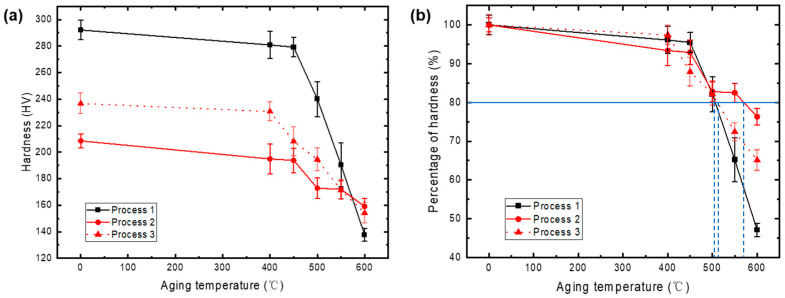
Curves of hardness (**a**) and hardness ratio (**b**) of Cu-Ni-Si alloy annealed at various temperatures for 60 min.

The alloys with three processes were subjected to isothermal annealing at temperatures ranging from 400 to 600 °C for one hour, with the resulting hardness variations shown in Figure 4a. It can be observed from the figure that as the annealing temperature increases, the hardness of all processed alloys decreases. Notably, for the CP alloy, the reduction in hardness becomes more pronounced at temperatures above 450 °C. After one hour of annealing at 600 °C, the hardness of the alloy was measured at 137.7 HV, representing a decrease of 154.42 HV compared to the as-fabricated state. The hardness of both DP alloys also decreased with increasing annealing temperature; however, the overall decline was more gradual compared to the CP alloy. After one hour of annealing at 600 °C, the hardness values for Process 1 and Process 2 were 159.1 HV and 154.2 HV, respectively, corresponding to decreases of 49.38 HV and 82.5 HV compared to the as-fabricated state. A comparison of the three processes reveals that the CP alloy exhibits the most significant reduction in hardness, indicating its inferior softening resistance, even though it underwent the least amount of cold deformation. It is generally understood that a higher degree of cold deformation facilitates recrystallization during annealing, leading to more pronounced reductions in mechanical properties.

The softening temperature curves for the alloys are presented in Figure 4 b, which indicates that the softening temperatures for the alloys are 505 °C (Process 1), 575 °C (Process 2), and 515 °C (Process 3), respectively. This suggests that, under similar deformation levels, DP alloys demonstrate superior softening resistance. This finding implies that the presence of discontinuous precipitation may inhibit the softening mechanisms within the alloys. Furthermore, it is noted that the softening resistance of DP alloys decreases with increasing cold deformation. To gain a deeper understanding of the softening behavior and mechanisms of these three alloys, it is essential to analyze the microstructural changes occurring during the annealing process.

**Figure 5:**
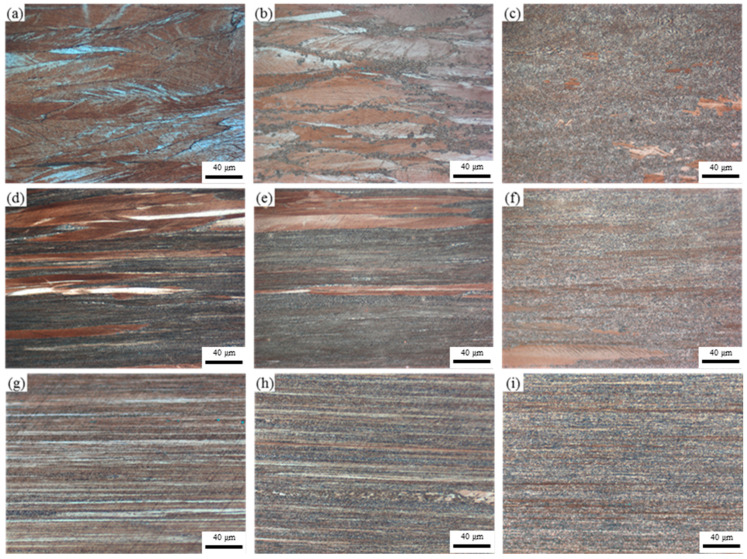
Optical microstructure of process 1 annealed at (**a**) 400 °C, (**b**) 500 °C, (**c**) 600 °C for 1 h, process 2 annealed at (**d**) 400 °C, (**e**) 500 °C, (**f**) 600 °C for 1 h, and process 3 annealed at (**g**) 400 °C, (**h**) 500 °C, and (**i**) 600 °C for 1 h.

Figure 5 presents microstructural images of Process 1, 2, and 3 during annealing at different temperatures. At lower softening temperatures, Process 1 retains a distinctly elongated microstructure, with grains elongated in the direction of drawing. However, due to the limited degree of deformation, the grains do not completely transform into a banded structure. Notably, significant deformation bands are observed within most grains, with angles between these bands and the drawing direction being less than 40°, as shown in Figure 5a. As the softening temperature gradually increases, a trend of decreasing deformation bands within the grains becomes evident, and new, finer microstructures begin to form near the grain boundaries, as illustrated in Figure 5b. When the annealing temperature reaches 600 °C, this finer microstructure gradually occupies the entire field of view within the grains, making it challenging to observe changes in the deformation bands, necessitating further analysis at higher magnifications.

In the DP alloys, a majority of the grains are occupied by discontinuous precipitates, and the grain shapes almost completely transform into a layered deformation structure, becoming more pronounced with increasing deformation levels. Due to the abundance of discontinuous precipitates within the grains, it is difficult to observe changes in the deformation bands, as seen in Figure 5d,g. As the softening temperature increases, it becomes apparent that numerous fine microstructures form between the layered structures of the DP alloys. These fine microstructures are similar to those found in the CP alloys, and their proportion increases with the level of deformation.

**Figure 6:**
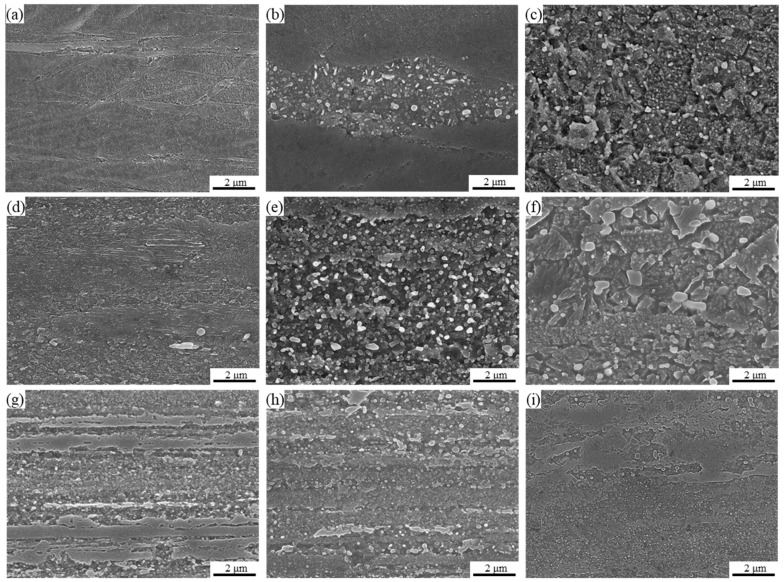
SEM of alloy 1 annealed at (**a**) 400 °C, (**b**) 500 °C, (**c**) 600 °C for 1 h, alloy 2 annealed at (**d**) 400 °C, (**e**) 500 °C, (**f**) 600 °C for 1 h, and alloy 3 annealed at (**g**) 400 °C, (**h**) 500 °C, and (**i**) 600 °C for 1 h.

Figure 6 presents SEM images of the microstructures of the three as-fabricated alloys. From Figure 6a, distinct deformation bands can be observed, which correlate well with the microstructure shown in Figure 5a. As the annealing temperature increases to 500 °C, discontinuous precipitates begin to appear near some grain boundaries. When the annealing temperature reaches 600 °C, it becomes evident that some CP phases have grown to several hundred nanometers. In CP alloys, both the precipitation of DP phases and the growth of CP phases contribute to a decrease in the mechanical properties of the alloy.

From Figure 6d–i, it is observed that in DP alloys, the morphology of the DP phase gradually transitions from an initially fibrous shape to a spherical shape as the softening temperature increases. Furthermore, with increasing temperature, these spherical precipitates grow larger, and this spheroidization phenomenon can be observed at lower temperatures with greater cold deformation of the DP alloys. The primary reason for this spheroidization is likely due to the effects of plastic deformation, which increases the dislocation density and distortion energy within the alloy, facilitating the dissolution and spheroidization of the lamellar precipitates during annealing, thereby leading to spheroidization annealing. This phenomenon may adversely affect the mechanical properties of the alloy. A more detailed analysis of the size and morphological changes of the precipitates in DP alloys will require additional TEM data.

## 4. Discussion

**Figure 7:**
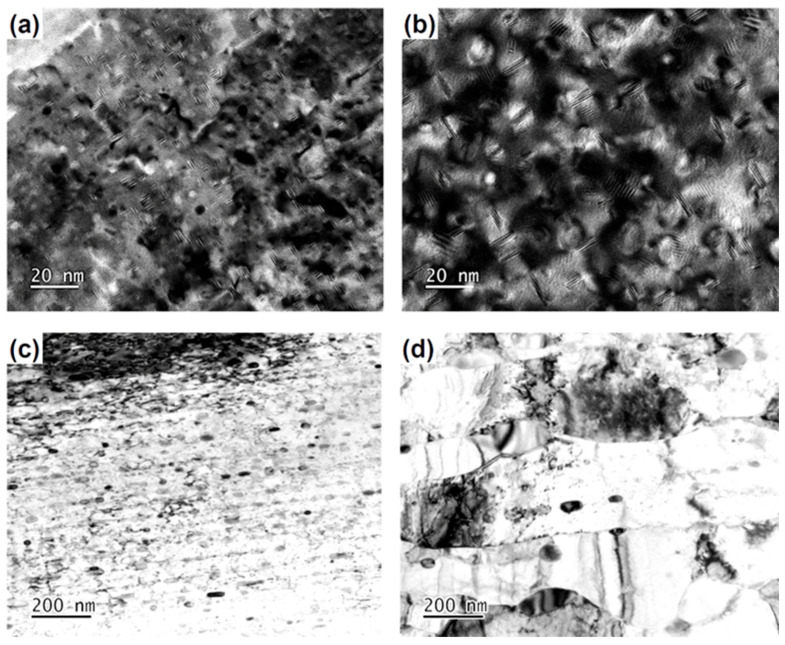
Transmission electron microscopy of Cu-Ni-Si alloys: the dark field images of the alloy containing CP annealed at (**a**) 400 °C, (**b**) 600 °C for 1 h, and the bright field images of the alloy containing DP annealed at (**c**) 400 °C, (**d**) 600 °C for 1 h, respectively.

To further investigate the changes in the morphology and size of the precipitates during the softening process of the alloys with two different precipitates, transmission electron microscopy (TEM) observations were conducted, as shown in Figure 7. From Figure 7a,b, it can be observed that in the alloy containing the CP, the proportion of CP was determined to be 42.27% using Matthiessen’s rule [50], by calculating the volume fraction (φ) of Ni₂Si precipitates based on conductivity changes during the aging process. The size of the precipitates coarsens with increasing annealing temperature during isothermal annealing. At an annealing temperature of 400 °C, the size of Ni_2_Si precipitates is less than 10 nm, and upon reaching 600 °C, the size coarsens to approximately 20 nm. In Figure 7c,d, it is clear that for the alloy containing DP, the proportion of DP reaches 100%, with no CP observed in the alloy. This indicates that DP fully dominates the microstructure at this stage. After annealing at 400 °C for one hour, the fiber-shaped discontinuous precipitates transform into a spherical morphology while still maintaining a linear arrangement. Additionally, no recrystallized grains were observed in the alloy. After annealing the alloy at 600 °C for one hour, the initially closely packed spherical precipitates were found to coarsen, with a significant increase in the distance between the precipitates, and new recrystallized grains were observed, with grain sizes exceeding 500 nanometers.

**Figure 8:**
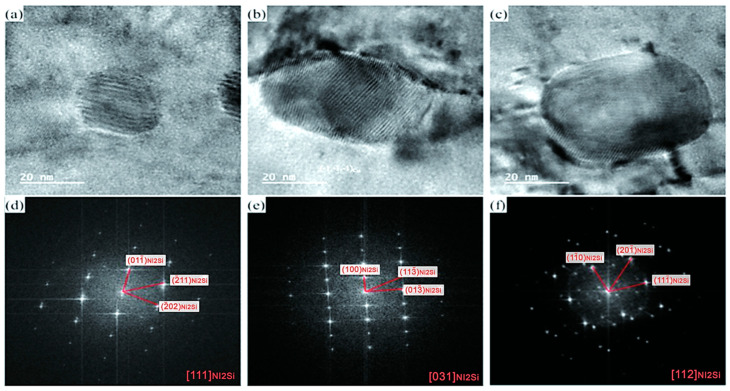
HRTEM images of the precipitated phases of the alloys containing DP annealed at (**a**) 400 °C, (**b**) 500 °C, and (**c**) 600 °C for 1 h and their corresponding FFTs (**d**) 400 °C, (**e**) 500 °C, and (**f**) 600 °C, respectively.

Using high-resolution transmission electron microscopy, the spherical phases of the alloy were observed at different annealing temperatures, as shown in Figure 8. It was found that the spherical phases increase in size with rising annealing temperature, and their morphology gradually transitions from spherical to ellipsoidal. This phenomenon can be attributed to the high stored distortion energy within the precipitates following severe cold deformation of the alloy, which promotes the melting and sphericity of the Ni_2_Si precipitates during annealing. Fast Fourier Transform (FFT) analysis of the HR-TEM reveals that the spherical phase is still the δ-Ni_2_Si phase, characterized by an orthorhombic structure with lattice parameters of a = 0.706 nm, b = 0.500 nm, and c = 0.373 nm.

**Figure 9:**
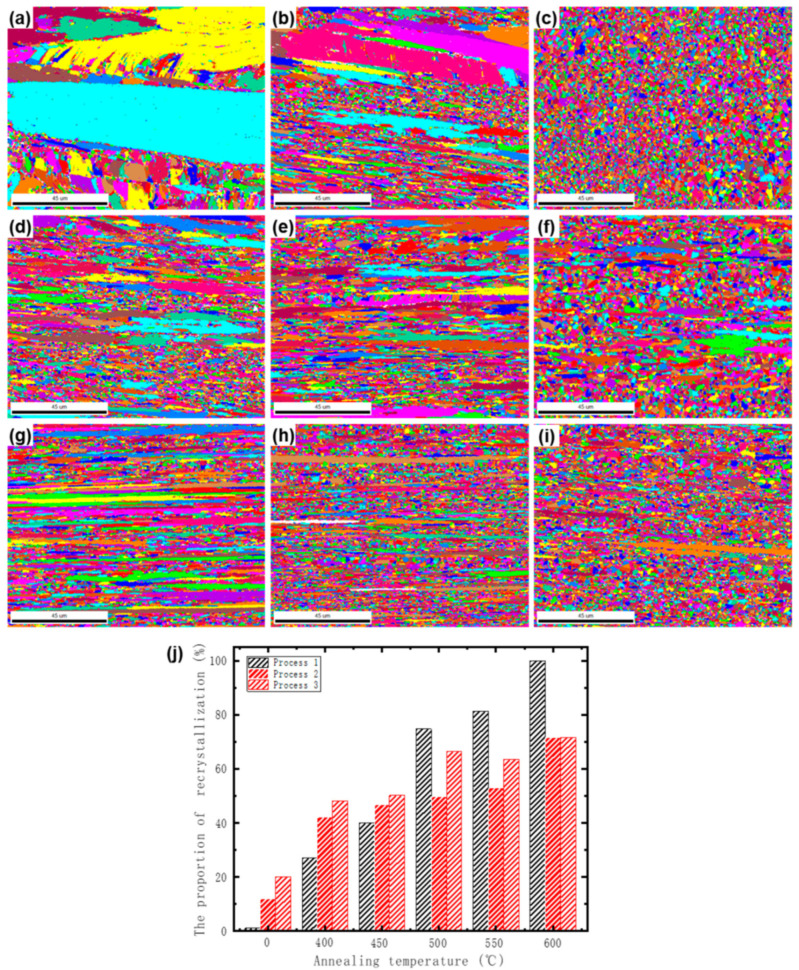
EBSD grain diagrams of process 1 annealed at (**a**) 400 °C, (**b**) 500 °C, and (**c**) 600 °C for 1 h, process 2 annealed at (**d**) 400 °C, (**e**) 500 °C, and (**f**) 600 °C for 1 h, and process 3 annealed at (**g**) 400 °C, (**h**) 500 °C, and (**i**) 600 °C for 1 h; Recrystallization volume fraction of alloy when annealed at different temperatures (**j**).

Figure 9a–i presents the EBSD of the alloy with three processes of annealing at different temperatures. The recrystallized volume fraction of the alloy was statistically determined using a grain orientation difference threshold of less than 0.8, as shown in Figure 9j. At an annealing temperature of 400 °C, all three alloys still exhibit a relatively intact deformed microstructure, characterized by numerous elongated grains, as shown in Figure 9a,d,g. Additionally, varying degrees of recrystallization are observed in all three processes, with Process 1 displaying the lowest degree of recrystallization while Processes 3 shows the highest recrystallization volume fraction.

When the annealing temperature is raised to 500 °C, the degree of recrystallization for all three processes increases, with Process 1 showing the greatest increase, followed by Process 3, while Process 2 shows a smaller increase, as illustrated in Figure 9b,e,h,j. At an annealing temperature of 600 °C, it is observed that Process 1 has essentially completed recrystallization, with equiaxed recrystallized grains present throughout the alloy and no elongated deformed grains observed. The degree of recrystallization in processes 2 and 3 also further increases compared to 500 °C, but does not exceed 70%.

The analysis indicates that the recrystallization degree of the alloy with CP is lower than that of the alloy with DP when annealed at temperatures below 500 °C. However, when the annealing temperature exceeds 500 °C, the recrystallization degree of the CPed alloy increases rapidly, surpassing that of the DPed alloy, and reaches complete recrystallization at 600 °C. In contrast, although the DP alloy begins recrystallization at a lower temperature, its recrystallization degree does not significantly increase with higher annealing temperatures; even at 600 °C, the recrystallization degree remains below 70%. This phenomenon suggests that discontinuous precipitation exerts a certain degree of suppression on recrystallization, resulting in a more stable recrystallization degree for the DP alloy during high-temperature annealing. This stability may be attributed to the obstructive effect of discontinuous precipitates on the movement of grain boundaries during the annealing process.

**Figure 10:**
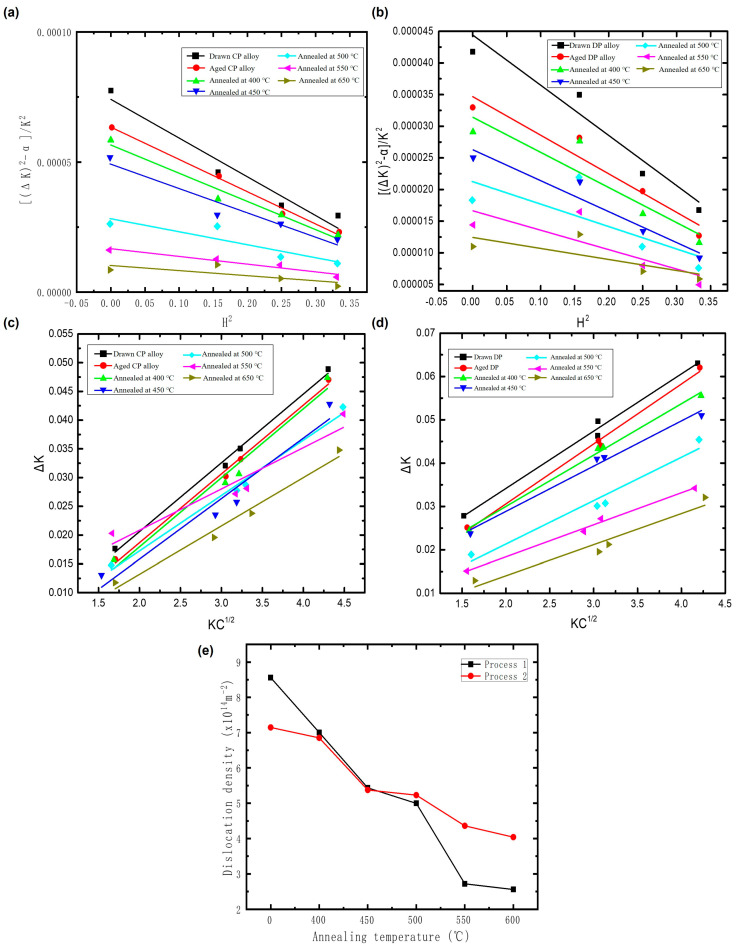
Fitted line plots of [(∆K)^2^ − α]/K^2^-H^2^ for process 1 (**a**) and process 2 (**b**), fitted line graphs of ∆K − KC^1/2^ for process 1 (**c**) and process 2 (**d**), and dislocation density (**e**) of process 1 and 2 after holding at different temperatures for one hour.

The dislocation densities of Process 1 and Process 2 at different annealing temperatures were calculated by the Modified Williamson-Hall method [51,52]. The calculated data were collected by XRD. The fitting lines for [(∆K)^2^ − α]/K^2^ − H^2^ and ∆K − KC^1/2^ are illustrated in Figure 10a,b and Figure 10c,d, respectively. The half-width of the peaks is closely related to grain size, microstrain, and defects, and it can reflect the defect concentration within the alloy matrix to a certain extent. In Figure 10, the vertical axis ∆K represents the half-width of the peaks. It is evident that as the annealing temperature increases, the half-width gradually decreases, indicating an overall downward trend in defect concentration. This suggests that the dislocation density of the alloy decreases progressively with rising annealing temperature.

The dislocation density of the two alloys at different annealing temperatures was calculated, revealing that the dislocation density for both alloys gradually decreases with increasing annealing temperature, as shown in Figure 10e. It is evident from the figure that the CP alloy exhibits a higher dislocation density after cold deformation. This is attributed to the smaller size of the precipitates in the CP alloy, which effectively pin dislocations and hinder their movement. In contrast, the DP alloy contains a significant amount of discontinuous phases within the matrix. However, the thickness of these discontinuous precipitates is relatively small, which prevents them from stablely accommodating dislocations. As a result, dislocations primarily reside within the matrix. The reduction in the volume fraction of the matrix subsequently decreases the capacity for dislocation accommodation, leading to a lower dislocation density in the DP alloy overall.

As the annealing temperature rises to 500 °C, the dislocation density of the CP alloy decreases significantly. This reduction is attributed to both recovery processes and the coarsening of the CP phase. The coarsened CP precipitates exert a diminished pinning effect on dislocations, allowing for easier dislocation motion. As a result, numerous dislocations of opposite signs can effectively cancel each other out, leading to a rapid decline in dislocation density. In contrast, the dislocation density of the DP alloy consistently decreases with increasing temperature throughout the softening experiments, although the rate of decline is relatively small. This suggests that the decrease in dislocation density in the DP alloy is somewhat inhibited. This inhibition primarily arises from the presence of DP phase boundaries, which can impede dislocation movement.

This study provides insights into the softening resistance behavior and mechanism of Cu-Ni-Si alloys with discontinuous precipitation (DP), contributing to the understanding of their application as high-current connectors in high-temperature environments. However, several future research avenues can further explore and enhance the findings of this work:

1. Advanced Alloy Development: The results suggest that Cu-Ni-Si alloys with DP phases exhibit superior softening resistance compared to those with continuous precipitation (CP). Future research can focus on optimizing the alloy composition by introducing microalloying elements or advanced processing techniques to further improve both mechanical strength and electrical conductivity, even at higher temperatures.
2. In-Depth Softening Mechanism Analysis: While this study elucidates the basic softening mechanisms for CP and DP alloys, more detailed investigations are required to fully understand the recrystallization behavior and the role of dislocation dynamics in DP alloys.
3. Industrial Applications and Long-Term Performance: One of the key motivations behind this study is the application of Cu-Ni-Si alloys in high-current connectors, particularly for electric vehicles and other high-power electronics. Future work should evaluate the long-term stability and durability of these alloys under real-world conditions, including cyclic thermal loading, mechanical stress, and environmental factors such as corrosion.

The results of this study provide a clear path for the future use of Cu-Ni-Si alloys with discontinuous precipitation in high temperature environments. The enhanced softening resistance demonstrated by DP alloys makes them highly suitable for critical applications where mechanical reliability and electrical performance must be maintained over extended periods. The findings can be directly applied to improve the design and production of high-current connectors used in electric vehicles, industrial machinery, and power distribution systems, thereby contributing to the advancement of more durable and efficient electronic systems.

## 5. Conclusions

This work studied the soften temperature, the microstructure evolution during soften, and the soften resistance mechanism of the Cu-6Ni-1.13Si alloys. Results are summarized as follows:

1. The alloy with continuous precipitation achieved a softening temperature of 505 °C after undergoing cold deformation with a 75% area reduction. In contrast, the alloy with discontinuous precipitation attained softening temperatures of 575 °C and 515 °C after experiencing 75% and 97.5% cold deformation, respectively.
2. In the alloy with continuous precipitation, the softening mechanism involves the gradual coarsening of the continuous precipitates. As these precipitates grow larger, their pinning effect on the grain boundaries weakens, leading to a higher degree of recrystallization in the alloy. Additionally, the coarsened precipitates exert a reduced pinning effect on dislocations, resulting in a significant decrease in dislocation density during the annealing process.
3. In the alloy with discontinuous precipitation, the higher softening performance can be attributed to the small interparticle spacing of the discontinuous precipitates after deformation. This spacing effectively hinders grain boundary migration, reducing the degree of recrystallization. Furthermore, the interfaces of the precipitates impede dislocation motion, leading to a more gradual decline in dislocation density. Additionally, the sphericity of the discontinuous precipitates further contributes to the obstruction of dislocation movement.

## Figures and Tables

**Table 1:** Comparison of the properties of CP alloy and DP alloy.

Alloy	Composition (wt.%)	Aging Time (Minutes)	DP Area Fraction (%)	Strength (MPa)	Conductivity (%IACS)
CP alloy	Cu-4.75Ni-1.13Si	30	0	678 ± 6.68	34.6 ± 0.34
DP alloy	Cu-4.75Ni-1.13Si	1440	100	552 ± 7.79	50.8 ± 0.02

## Data Availability

Data is contained within the article.
